# Social media ostracism and creativity: moderating role of emotional intelligence

**DOI:** 10.1186/s40359-024-01985-2

**Published:** 2024-09-13

**Authors:** Muhammad Waqas Amin, Jiuhe Wang

**Affiliations:** School of Economics and Management, Yanshan University, Qinhuangdao, China

**Keywords:** Social media ostracism, Emotional intelligence, Psychological rumination, Psychological safety, Creativity

## Abstract

The goal of this study is to learn more about social media ostracism, a stressor associated with online social networks, defined by feelings of rejection, exclusion, or ignoring. We investigate the connection between social media ostracism and worker creativity. We suggest that psychological safety and psychological rumination serve as intermediaries in this relationship. Furthermore, we investigate emotional intelligence as a relationship regulator. To verify our hypothesis, we gathered data with the help of the HR department from 244 workers of nine Chinese organizations. Our research shows that psychological rumination and social media exclusion are significantly correlated, but only in workers with low emotional intelligence. Furthermore, for individuals with strong emotional intelligence, we did not discover a statistically negative association between psychological safety and social media exclusion. Findings suggest that psychological safety and psychological rumination serve as mediating factors in the relationship between employee creativity and social media exclusion. This study illuminates the negative aspects of social media ostracism and reveals how it might hinder creativity. It also emphasizes how emotional intelligence functions as a moderator. Organizations may learn a lot from this study on how to lessen the negative impacts of social media exclusion on employee creativity.

**Supplementary Information:**

The online version contains supplementary material available at 10.1186/s40359-024-01985-2.

## Introduction

Social media usage in the workplace and its outcomes become a hot topic for researchers. Individuals use social media to communicate, interact, share information, and become a part of his/her favorite social communities [1–3]. Many modern organizations encourage their employees to use enterprise social media (ESM) for knowledge sharing, collaboration, and receiving information from other employees within the organization, considered a primary contributor to the success, competitiveness, and growth of the organization [4, 5]. Although social media use has positive effects, Social media is repeatedly exemplified by numerous stressors. That is, scholars investigated cyberbullying and information overload as prominent stressors [6]. As more individuals engage in online interaction and social media use, the potential for experiencing exclusion in these virtual spaces has increased. Understanding the impact of social media–based ostracism on creativity is crucial, as creativity is a vital driver for innovation, problem-solving, personal growth, and creating robust, novel, and valuable ideas at the workplace [7]. Employees’ creativity is considered a critical component of innovation, which encompasses both the invention of the latest ideas and their accomplishment [8]. The existing literature has primarily focused on the general effects of ostracism ( family ostracism, workplace ostracism) on various psychological and behavioral outcomes [9, 10]. Understanding the connection between SMO and employees’ creativity is supremely significant in the digital age. However, the impact of SMO on employees’ psychology and cognitive approach has been overlooked. This represents a significant research gap that warrants further investigation. Predominantly, we focus on the role of social media stressors (SMO) on employee’s behavior and creativity. The prominence of SMO relates in a way like, that organization must realize, how it can help or hinder creativity.

Research has repeatedly backed the idea that social media stressors have a detrimental effect on users. Organizations appear to be experiencing a revival in ostracism. SMO refers to the act of excluding someone in a social setting [11]. It can lead to feelings of social rejection and loneliness. Ostracism, however, is thought to be a phenomenon that exists everywhere and in a variety of circumstances [12]. As a result, previous research has revealed that Internet users experience cyber-ostracism. However, given that ESM can be used as a tool for employees to collaborate on projects, share information, and ask questions, thereby fostering teamwork and innovation [5, 13, 14]. Employee perception of social media usage can be influenced by SMO. If an employee frequently experiences exclusion or negativity on social media, it can affect their self-esteem, overall job satisfaction, and perception of their employer or colleagues. They may feel isolated, unappreciated, or less motivated in their work. This can have negative effects on individuals’ psychological well-being, including a decrease in self-esteem and an increase in negative emotions. Therefore, we concentrate on SMO and also define it, as the degree to which social media users think that they are excluded, redundant, or ignored by their social media friends. Yet, scarce studies have examined the linkage between SMO and employees’ creativity. Unfortunately, this study gap exists given the importance of stressors related to ESM in the workplace [15]. The mechanism by which SMO hinders employees’ capacity for creativity remains undermined. Hence, scholars urged for additional research on social media-related stressors, as well as their impact associated with the workplace [14]. Hence, an investigation of the link between SMO and employee creativity will aid in the foundation of managerial implications as well as the growth of theory.

Our study deals with the issue of social media stressor namely SMO. We squabble that different types of social media stressors have different effects on employees’ behavior. In the notion of ego depletion theory, this study is to examine the mechanism involving between SMO and creativity. Ego depletion theory according to [16] “a temporary reduction in the self’s capacity to engage in volitional action caused by the prior exercise of volition” (p. 1235). Ego depletion was first introduced by social psychology. Numerous domains, including personality, consumer behavior, cognitive psychology, decision-making, and organizational behavior, continue to apply and explore them [17–19]. It describes the circumstance in which an individual performs worse on self-control tasks after having completed a self-control-requiring task in the past. According to the theory, future regulatory efforts becoming more and more challenging as resources are depleted. As a result, the more regulatory duties individuals must undertake throughout the day, the more tricky it is for them to retain effort, tenacity, and, eventually, appropriate performance on a variety of jobs. Moreover, such depleted individuals are also more inclined to deceive and misread organizational performance, mislead and undermine coworkers, and be vocally aggressive toward colleagues, supervisors, and subordinates [20]. Hence our research projected that the exhibition of SMO provides a foundation for psychological cost, like, psychological safety and psychological rumination, which limits employees to present creativity in their job tasks.

From the above argument, a potential inconsistency emerges. We further move forward to study such circumstances in which SMO is interconnected with psychological safety, psychological rumination, and creativity. The value of resource depletion is not the same for all individuals [20]. However, this condition may not apply to all individuals. People do have diverse personalities, and depending on the circumstances, they act in different ways. So, a person’s reaction to stress is determined by their traits. Although depilation of individual resources due to SMO always prevails, individuals having high emotional intelligence may stay away from the adverse effect of recourse depletion. Emotional intelligence comprises the ability to understand and manage emotions effectively and the flexibility to acclimatize unpredicted situations [21]. Individuals with higher emotional intelligence may be better equipped to cope with the negative emotions associated with social media stressors such as SMO [11] and are expected to tolerate a smaller amount of resource loss when dealing with SMO., in turn, we propose that emotional intelligence can moderate the significant affect of SMO on psychological rumination, psychological safety, and indirect negative impact of SMO on employees creativity passing through psychological rumination and psychological safety and allowing individuals to maintain their creativity levels.

The ongoing study aims to examine the relationship among social media stressors such as (SMO), psychological states including (psychological rumination, and psychological safety), and creativity through a survey of Chinese employees, contributing to the existing literature in various important ways. Firstly, adding to on-hand literature on ESM and social media stressors by incorporating it with ostracism literature. Secondly, our research is the primary effort to look at how social media stressors—specifically, SMO—affect employees’ creativity. In this approach, we help widen the scope of social media stressors and their effect on workers’ creativity. Thirdly, we integrate SMO with ego depletion theory. By doing this, we acquire tools such as the depletion of resources (self-control) and identify psychological safety and rumination as a mechanism through which SMO is allied to creativity. Fourthly, we implement a contingency view and introduce emotional intelligence as a significant edge on SMO. Finally, this study adds to the literature on ego depletion theory by incorporating and experimentally assessing its function in propagating the blow of SMO on creativity.

The following segment of the study begins with a review of pertinent literature. Following that, three mediating and two moderating assumptions are provided. Section 3 is highly structured on our study technique, including data gathering methodology, and sample demographic information, in addition to constructing operationalization. The findings of the data analysis are presented in Sect. 4. Section 5 reviews the findings, gives theoretical and practical suggestions, and defines the study’s limitations. Section 6 concludes this research.

## Theoretical background and hypothesis development

### Social media ostracism

Ostracism refers to the act of excluding an individual or a group from social acceptance, inclusion, or participation [22]. Researchers argued that ostracism commonly prevails in social settings including social media [11]. Social media is used to connect, interact, share ideas, contact, and knowledge sharing among employees [23, 24]. When coworkers on social media platforms shun others by not responding to their messages, neither seeing nor appraising and giving likes to their postings, or acting as, if a person is not a member of their organization’s social media network, it can cause SMO for employees.

Ostracism tends to in result adverse consequences as it causes a sense of " social pain” [25]. The theoretical background of ostracism can be traced to various psychological theories and frameworks. One prominent theory is the human need theory, which posits that humans have basic psychological needs necessary for their well-being and functioning. According to this theory, developed by [26], the need for belongingness and social acceptance is fundamental to human nature. Ostracism on social media platforms can often take the form of cyberbullying [27]. When individuals are deliberately excluded from online activities or targeted by negative comments, and messages, they can experience emotional distress and harm to their self-esteem. It is very imperative to note that while social media can be a platform for ostracism [11], it can also serve as a tool to address and combat it. By promoting positive online behavior, encouraging empathy and inclusivity, and actively working on creating safe and supportive digital spaces, individuals and platforms can diminish the pessimistic impact of ostracism on social media. SMO refers to the act of shunning individuals within an online platform [11, 28]. This can happen through various means, including blocking, or excluding someone from conversations, groups, or events. SMO can have significant effects on individuals’ well-being and sense of social belonging. Given the nature of ostracism, we discover numerous aspects of SMO. Like, Due to their hectic schedules, social media users generally don’t reply to other users’ comments. Irrelevant connections could be the cause of social media exclusion. That instance, if employees post about hedonistic events on ESM, other users are likely to ignore the posts. A person’s sense of connection to their connections is jeopardized by being ignored. Hence, SMO is a complex phenomenon that people might encounter and it can be challenging to grasp how it starts and ends, thus it is a part of everyday life for social media users. Employees try to build and sustain interpersonal connections with their colleagues on social media to get the maximum payback of social media for job performance [29, 30], and fulfillment of their social needs projected by social media customers [31]. Consequently, ostracism from social media contacts leads to a disruption in the individual’s needs, thoughts, and emotions, triggering various cognitive and emotional reactions. However, the intuition of SMO is not essentially to ruin workers [32]. It is just an exemption of optimistic commitment in online networks eventually it is apposite in the social media context [11]. Although SMO is treated as a trifling issue, it has an important effect on ostracized employees’ psychological emotions and cognitive behavior [11]. In the light of ego depletion theory [33], for example: targeted personnel, who are perceived to be ignored, avoided, or rejected by other colleagues on social media, are more likely to feel psychological pressure, discomfort, and less work engagement resulting in insignificant creativity. Furthermore, staff use social media to intermingle, and exchange information as well as to communicate with others to fulfill their needs through interpersonal exchange. However, for ostracised employees, the use of social media creates an intimidating environment, resulting in psychological pressure and insignificant creativity.

### Creativity

The majority of research has been done in behavioral labs and has been framed from an “intrinsic motivation” perspective. This viewpoint contends that an individual’s intrinsic motivation is affected by the situation in which they accomplish a task, which in turn affects how creatively they can express themselves [34]. Additionally, creativity has been categorized into two main dimensions: originality and effectiveness. The former focuses on the novelty and uniqueness of ideas, while the latter assesses their value and utility in addressing a problem or meeting a goal [3, 35]. Numerous cognitive abilities have been found to influence creativity, such as high levels of fluency, flexibility, and originality of thinking. The ability to generate a large number of ideas and to think beyond conventional constraints contributes significantly to creative output. Intrinsic motivation, driven by genuine interest and enjoyment, is found to be crucial for initiating and sustaining creative endeavors. Extrinsic motivators, such as rewards and recognition, may also influence creativity, but their effects are more context-dependent. Employees are thought to be most creative when they have a high level of intrinsic motivation—that is when they are passionate about a professional activity and want to engage in it for the sake of the activity itself [36–38].

Social media provide a platform for social interactions, collaborative environments, and diverse perspectives that can enhance creative thinking by fostering the exchange of ideas, challenging assumptions, sharing knowledge, and promoting interdisciplinary approaches [39]. Social media used by employees in the organization ( ESM ) is different from public social media(PSM). ESM platforms have become increasingly popular tools for collaboration and communication within organizations. ESM use has been found to positively influence individual creativity [40]. Studies highlight that these platforms provide opportunities for idea generation, knowledge sharing, and informal discussions, where cognitive resources can be quickly and conveniently accessed [41]. Collaborative features of these platforms facilitate the exchange of diverse perspectives, enhancing the likelihood of new and innovative ideas. Research also suggests that social interactions on ESM platforms increase intrinsic motivation, helping employees overcome creative blocks and fear of criticism [41–43]. Employees are bound to join ESM at any cost, while users on PSM are free to use or switch to other platforms. Employees can share hedonic stuff, and information with colleagues on the ESM platform [44, 45]. Meanwhile, PSM provides a wide range of social, cognitive, and hedonic activity platforms, to build social associations, sharing photos and videos with social media users [46, 47]. Thus, ESM compelled employees to interact with organizational members having competing interests. Hence, conflict of interest my lead employees keep silent on ESM, resulting in not giving a response to any post or message and deliberately not taking part in any routine discussion. Thus, ignoring others may lead to psychological stress, and insecurity and affecting work engagement [48]. However, the emergence of SMO – the deliberate exclusion or rejection of individuals within these online communities – has raised concerns about its potential impact on one’s creativity [49]. Employees are less likely to contribute creative ideas in performing their tasks when they experience rejection. To the paramount of our knowledge, none of the studies has been carried out to investigate employees’ creativity using social media stressors. Hence, our motivation behind this unique study is to investigate how employees’ creativity is being affected by social media stressors namely SMO.

### Ego depletion theory

Ego depletion theory, also known as self-control theory, was developed by psychologists [50]. The supposition postulates employee self-control or willpower resources are limited and can easily be depleted with repeated or prolonged use, resulting in a decrease in the employee’s ability to regulate oneself effectively, suggests that when individuals engage in acts of self-control, such as resisting the temptation or suppressing emotions, their self-control reserves become diminished, leading to reduced employees self-control performance in subsequent tasks [51, 52]. For instance [50], found that participants who initially resisted eating tempting cookies performed worse on an unsolvable puzzle task compared to those who did not resist temptation. These findings suggest that self-control depletion affects cognitive resources needed for subsequent self-control tasks. This theory has been extensively studied and researched in the field of psychology [53].

The concept of ego depletion is rooted in the psychodynamic and cognitive approaches to employees’ behavior. According to [54] psychoanalytic theory suggests that the self is tranquil of three parts: the id, ego, and superego. The ego, being the rational part, is responsible for self-control and decision-making. Building upon this idea, ego depletion theory proposes that the self-control capacity is a central aspect of employees’ behavior, contributing to the regulation of emotions, impulses, and thoughts [50]. Explain the foremost efficacy of ego depletion theory: Resources gained from personnel and social activities are well sheltered and appropriate. Personal resources include a sense of self, personal characteristics, and energies. Meanwhile, social resources take account of social support, which comes from communication, relation, and interaction with other groups or individuals [55]. Individuals make an effort to acquire, hold onto, and preserve their social resources because they are the primary route to acquiring valuable personal resources. According to the ego depletion theory, when there is a possibility of losing resources, people would experience psychological discomfort. An individual who practices ego depletion tends to exhibit cognitive biases, miscalculate their ability, and feel a weakened sense of self-control over the future [56]. Depleting cognitive resources impairs individual creative performance. Individuals are less successful at reacting promptly when resources (limited) run out, even in an apparent unconnected domain of action. Ego depletion can discourage self-regulation, leading to impulsive decisions and a decline in creative performance. Numerous studies show that depleted individuals cannot carry out daily tasks and respond in a broad range as they would be able to if not depleted. Once depleted, workers’ ability to show self-control or reveal proper behavior based on their limited resources befalls challenging, resulting in inappropriate conduct [17]. Therefore, individuals under stressful conditions play defensively to protect their emotional, social, and cognitive resources.

**Fig. 1:**
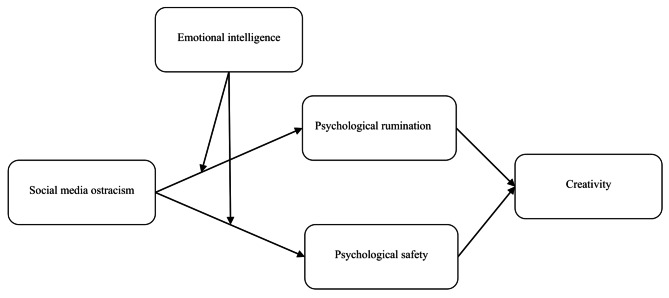
Conceptual and hypothesized model

In addition, ego depletion theory suggests that the impact of a stressor differs from person to person depending on their attributes and characteristics. Moreover, individuals give more value to their social connections and if they are unable to receive perceived social care, thus, this situation has an unfavorable impact on individuals’ emotional and psychological state. Consequently, social media stressors like SMO have a negative impact on individuals’ psychological, emotional, and cognitive states [57]. When employees experience ostracism on social media, it can deplete their psychological and cognitive resources. In light of ego depletion, susceptible willpower resources are depleted [50]. Emotional intelligence may support individuals to trim down the adverse effect of stressors on employees’ psychological discomfort state (rumination and safety) and respectively on employees’ cognitive approach. We argue based on ego depletion theory, that SMO may linked with individual psychological discomfort state (rumination and safety), and affect individual creativity. Meanwhile, the depressing effect of SMO may be moderated by emotional intelligence. Fig. 1 shows the conceptual model.

### Social media ostracism, psychological rumination, and psychological safety

Social media within the workplace can be used as a tool for employees to collaborate on projects, share information, and ask questions, thereby fostering teamwork and innovation. Employees can use ESM platforms to connect with colleagues and industry professionals, which can enhance their networking opportunities and career growth prospects [40]. SMO refers to the exclusion or isolation of individuals on social media platforms. This can be a result of various factors, such as not receiving likes or comments on posts, being ignored by social media groups or conversations, and being targeted by negative or hurtful comments [58]. Employee perception of social media usage can be influenced by SMO. If an employee experiences frequent exclusion or negativity on ESM, it can affect their self-esteem, overall job satisfaction, and perception of their employer or colleagues. They may feel isolated, unappreciated, or less motivated in their work [11, 59]. When individuals experience SMO, such as being excluded from enterprise online social groups or being subject to cyberbullying, the end product can be psychologically struck personally (i.e. psychological rumination, psychological safety). Psychological rumination refers to the repetitive thinking about negative experiences, which can have detrimental effects on mental well-being. Rumination can further intensify feelings of social exclusion and negatively impact overall psychological functioning [60]. Psychological safety refers to an individual’s perception of safety within a group or social environment, where one feels free to express oneself without fear of negative consequences such as rejection or humiliation [61].

When someone experiences SMO, it can have a profound impact on their mental and emotional well-being. Being excluded or ignored on social media may trigger feelings of sadness, loneliness, and low self-esteem [11, 62]. These negative emotions can then lead to psychological rumination and a decrease in psychological safety, where individuals excessively think about the event, replaying it in their minds and analyzing what they might have done wrong. This means that individuals who experience more frequent instances of SMO are more likely to engage in greater rumination and decrease psychological safety. The constant exposure to social media interactions, where exclusion or negative feedback is prevalent, may contribute to a heightened focus on negative experiences, leading to an increase in psychological rumination and a decrease in psychological safety. When individuals repeatedly encounter ostracism on social media, it may erode their sense of belonging, acceptance, and value in their online communities. This can result in heightened feelings of insecurity, anxiety, and a lower sense of psychological safety. The fear of further SMO may prevent individuals from freely expressing themselves or fully engaging in online enterprise social interactions [63], limiting their participation and potentially negatively impacting their mental health [64], it may lead to a decrease in their level of psychological safety. Humans have a fundamental need for social belonging and inclusion. According to ego depletion theory [65], When individuals are excluded on social media platforms, it can lead to negative psychological consequences including feelings of sadness, anger, loss of self-control, and lower self-esteem decrease in psychological safety. Hence, SMO have psychological effect on employees. Thus, we propose that.

#### H1

Social media ostracism is positively associated with the psychological rumination of social media users.

#### H2

Social media ostracism is negatively associated with the psychological safety of social media users.

### Linking psychological rumination, psychological safety to creativity

SMO spoils individuals’ valuable psychological and self-control resources, thereby rooting psychological rumination and decreasing psychological safety. Ego depletion theory predicts employees whose self-control and self-regulatory resources have been depleted, lead to inappropriate behavior [66], self-control requires mental effort. When individual employees’ limited self-control resources are depleting they get psychological pressure. Accordingly, this study argues that psychological rumination and a decrease in psychological safety caused by SMO affect employees’ creativity.

According to [67], Creativity is stated as providing novel, valuable ideas and solutions for any problem. It is influenced by various factors such as psychological processes and the external environment. Furthermore, cognitive processes involve creative thinking. Hence, various mental processes (i.e. problem framing, analogical reasoning, flexibility, and divergent thinking) arise in creativity [35, 68]. Moreover, social factors such as cultural norms, social interactions, and environmental conditions (organizational environment) foster creativity. Creative ideas emerge through employee interactions, brainstorming, collaborations, and exposure to diverse perspectives and ideas [8]. Consequently, it is noted that creativity is an emergent property of complex systems, that arises from interaction of different social, environmental, and cognitive factors. When an individual is psychologically, mentally, socially, and environmentally stable, can be a creative thinker and generate novel ideas, and solutions for problems. In this current perspective, we use ego-depletion theory to enlighten employees’ creativity.

Employees’ psychological and emotional resources are affected by SMO, thereby stimulating psychological rumination and affecting individuals’ psychological safety. Ego depletion theory indicates that individuals whose resources are depleted are less likely to feel confident, relaxed, and happy [69] therefore, Employees always have negative thoughts and insecurity. Hence, individuals who engage in persistent rumination are more likely to experience cognitive and emotional burdens that could hinder their creative abilities. The excessive focus on negative thoughts and emotions may restrict cognitive flexibility. Furthermore, when individuals feel psychologically safe, they are expected to take risks, share ideas, and think innovatively. Thus, SMO drags employees into a depressing psychological and emotional situation, thereby messing up their cognitive creative approach. Hence we proposed the following hypothesis.

#### H3

Psychological rumination is negatively associated with the creativity of social media users.

#### H4

Psychological safety is positively associated with the creativity of social media users.

#### H5

Psychological rumination and psychological safety will mediate the association between social media ostracism and employees’ creativity.

### Moderating role of emotional intelligence

Emotional intelligence is the aptitude to identify, understand, and deal with one’s own emotions as well as the emotions of others [21]. It is crucial in regulating individuals’ responses to various social situations [70] such as SMO. Individuals with higher levels of emotional intelligence may be better equipped to regulate their emotions and engage in adaptive coping strategies when faced with negative thoughts and emotions. Emotional intelligence influences how individuals greet, and consider any event that takes place in their routine lives [71]. This includes staying calm under pressure, managing stress, and handling conflicts or difficult conversations with emotional maturity. Conversely, individuals having weak emotional intelligence will bear high pressure.

According to ego depletion theory, the depletion of self-control, willpower, and decision-making resources differs from person to person [72]. Individuals having different traits, respond differently to their loss of limited recourses [11]. However, in our context Employee suffering from SMO may identify different degrees of psychological rumination and psychological safety because of their different level of emotional intelligence [73]. Therefore SMO plays a vital role in diminishing employees ' control over resource loss, employees who have high emotional intelligence are less likely to welcome ostracism effects or immediate recovery from resource loss. However, workers with high emotional intelligence are well equipped to handle the adverse effects of SMO, are more likely to prevent themselves from leading to rumination, and maintain their sense of psychological safety. On the other hand, workers with low levels of emotional intelligence are more susceptible to the negative shock of SMO. Indeed, emotional intelligence overcomes stressful conditions like SMO [74].

Based on a previous study, employees face negative behavior and events on social media [14]. Besides personality traits, every social media user faces psychological frustration encountered by SMO, including stressful situations and negative emotions [75]. However, individuals with higher levels of emotional intelligence can be better equipped to regulate their emotions and engage in adaptive coping strategies when faced with negative emotions and stressful situations [74]. As a result, they are more resilient to the harmful effects of rumination and psychological well-being. For that reason, when individuals have strong self-control over emotions, a sense of psychological frustration cannot lead to psychological rumination and a decrease in psychological safety. Based on ego depletion theory this study argues that emotional intelligence enhances workers’ capability to deal with SMO. Consequently, we assert that individuals with high emotional intelligence are better prepared to navigate and minimize the negative effects of SMO. Moreover, experience low psychological frustration, thereby decreasing the adverse consequence of SMO on psychological rumination and psychological safety. So, we proposed.

#### H6

Emotional intelligence moderates the positive association between social media ostracism and psychological rumination, such that when the emotional intelligence level is high, the positive relation is weaker and when the emotional intelligence level is low, the positive relation becomes strong.

#### H7

Emotional intelligence moderates the negative relationship between SMO and psychological safety, such that when the emotional intelligence level is high, the negative relation is weaker and when the emotional intelligence level is low, the negative relation becomes strong.

## Methodology

### Sample and data collection

To evaluate our hypothesis for this study, we created a questionnaire. We took up an online survey methodology to achieve a maximum and diverse sample response rate. China is rich in using social media so we conducted this study in China. According to a foremost professional consulting company, Towers Watson’s report states ESM is widely used by Chinese employees, accordingly 49% of respondents claimed ESM provides a platform to establish a sense of belonging, interaction, and communication because of its cost-effectiveness [76].

We further collaborated and contacted several companies’ HR managers. The backbone of every company is the HR department and HR managers are much more familiar with the company’s routines, and policies and also have employees’ personal and performance records. Initially, we chose 15 companies and explained our research purpose (i.e. creative performance of employees using ESM ). As such, among these 15 companies, 9 companies are willing to participate in our research work. Further, we made a promise to share the final report of our research and provide them with suggestions once we finished our research. We further, conducted random interviews of several selected companies’ employees before distributing the questionnaire. The purpose of the interview is to investigate whether or not employees of these companies utilize social media to interact and converse with their coworkers. Therefore, it is been confirmed that employees employ social media to be in touch with both internal employees and external customers.

We designed and generated an online survey link and distributed it to the targeted sample companies’ workers with the help of the HR department. HR department’s collaborative attitude generally increased the response rate. After three weeks, 301 responses were received, out of them 57 responses were incomplete and inaccurate so, discarded, finally we got 244 useful responses. By employing a chi-square test to match the first and last 25% of respondents on all variables, we were able to evaluate potential non-response bias [77]. No significant difference has been reported, signifying that non-response bias for this research was not a serious concern. Nevertheless, it’s very important to note that the cooperative behavior of the HR department may have direct to optimistic bias towards social media use amongst respondents. To address this issue we took three initiatives. Firstly, we established our study ambition with HR executives, as we would like them to understand our intention for this study was to find out whether and how social media usage manipulates employees’ creativity, rather than give support to the significant effects of social media. All of them agreed and verified similar goals. Secondly, managers only helped to regulate our online survey link within the organization and identified it as a university research project. Thirdly, we mentioned at the start instant of a questionnaire for better understanding that this questionnaire was established to familiarize how ESM users perform creatively. Furthermore, the secrecy of this survey was highlighted. Table 1 shows the sample’s demographic information.

**Table 1:** Demographic information

Measure	Items	Frequency	Percent	Measure	Items	Frequency	Percent
Gender	Male	160	65.57	Age Range	18–30	122	50.00
	Female	84	34.43		31–45	96	39.34
Education Level	High school	95	38.93		46 or above	26	10.66
	Undergraduate	116	47.54	Usage frequency	Hourly	34	13.93
	Masters or above	33	13.52		Several times 1 day	63	25.82
Number of ESM friends	50 or less	18	7.38		Once daily	73	29.92
Industry Type	51–100	77	31.56		Several times a week	58	23.77
	101–150	84	34.43		Once a week	16	6.56
	151–200	51	20.90	Usage experience	1 year or less	37	15.16
	201 or above	14	5.74		1–5 years	120	49.18
Job type	Marketing & Sales	40	16.39		5–10 years	74	30.33
	Accounting & Finance	70	28.69		above 10 years	13	5.33
	Human resource	49	20.08		31 or above	34	8.90
	Production and operations	85	34.84				

### Measures

All of the construct items were taken and measured from the existing literature to increase the reliability of empirical findings [78–83]. All the respondents of this study were Chinese, so to cope with the language barrier we were required to translate our English questionnaire into Chinese. Finally, we requested two Chinese native speakers, proficient in English, but they are not a part of our study but to help us translate our English questionnaire into Chinese language. To check the accuracy of the translation we further invited two experts, unfamiliar with the original English version questionnaire and they translated the questionnaire back into English. No systematic error was found between the original and English-translated versions. Hence, we considered the Chinese questionnaire an accurate reflection of the original English questionnaire to test our constructs. Furthermore, for a better understanding of respondents, all the possible descriptions of constructs were briefly explained in the questionnaire. We used a 5-point Likert scale arranged from 1 " strongly agree " to 7 " strongly disagree " to measure all items, except one item used a 4-point Likert scale arranged from 1 “never” to 4 “always”.

Measurement items for SMO were adopted by the ostracism scale established by [11]. Scholars already adopted this scale in the Chinese context [11, 84]. The scale was initially designed to measure ostracism at the workplace, this current study demands to modify workplace ostracism items in a social media context. Respondent rated ten items under SMO. A psychological safety measurement five-item scale was adopted from [79]. We modified the items by just changing “team” to “organization”. Psychological rumination was measured by a 5-item Ruminative response scale. It is a rating scale developed to evaluate people’s behavior and thoughts when they are depressed [82, 83]. To measure emotional intelligence, measurement sixteen-item items scale was adopted from [85]. Four-item scale from [81] was used to measure employees’ creativity. The related literature for survey items is précised in Appendix A. Several variables have been controlled that can affect this study’s results. This is followed by previous social media studies to control various variables that are demographic traits (i.e. age, gender, and education ) [11, 86, 87], usage frequency (i.e. hourly, daily, or weekly ) [87], users familiarity and several friends also affects employees behavior ( below 5 years, 5–10 years, 11–15 years, 16 years or above) [87], several contacts on ESM ( under 100, 101–200, 201–300, 301–400, 401 or above) [88], Nature of job may impact on workers social media usage, for instance, accounting professional use less social media than sales and marketing professionals [89].

## Data analysis and results

### Common method bias

The entire data collected for the in-progress study is self-reported, resulting there might be a chance for common method bias. Checking the possible severity of common method bias we used several methods. First, Harman’s single-factor test was carried out. Results showed that common method bias was not an issue for our data, neither a single nor a general factor reports the variance < 50%. Secondly, confirmatory factor analysis pointed out that five-factor model suggests better fit [(X2 (714) = 1552.921, Tucker–Lewis index (TLI) = 0.94, comparative fit index (CFI) = 0.94, and root-mean-square error of approximation (RMSEA) = 0.07)] than a single factor (X2 (724) = 5392.758, TLI = 0.65, CFI = 0.68, and RMSEA = 0.16). These results indicate that no possibility of common method bias was found in our data. Third, significant moderating effects of emotional intelligence, indicated that data is free from common method bias. Collectively, the result shows that in our data common method bias is not a significant issue and also strengthens the legality of our findings.

### Measurement model

The scale’s convergent validity, discriminant validity, and reliability were assessed using confirmatory factor analysis. The satisfactory fit between the measurement model and date base was declared in the CFA result (χ2 = 1552.921, d.f. = 714, TLI = 0.94, CFI = 0.94, and RMSEA = 0.07). Table 2 shows that all the item loading is higher than the suggested benchmark of 0.50. To test the convergent validity and composite reliability of constructs, Cronbach’s alpha and average variance reliability (AVE) are been assessed. The table shows that the values of composite reliability and Cronbach alpha for each construct are higher than the benchmark value of 0.70 and the AVE score is also ranged from 0.57 to 0.81. Thus, the result indicates that the convergent validity is satisfactory.

Discriminant validity is appraised by comparing the square root AVE and correlation for each construct [1, 90]. Discriminant validity requirements were also satisfactory. Table 2 illustrates that the square root of AVE is greater than the correlation between each construct. Therefore, the measurement model is obsessed with adequate reliability, convergent validity and discriminate validity.

**Table 2:** Reliabilities and correlations

Variable	Mean	Std. Deviation	CR	AVE	Loadings	1	2	3	4	5	6	7	8	9	10
1. Social media ostracism	5.13	1.44	0.97	0.79	0.78 − 0.95	**0.89**									
2. Psychological safety	3.08	1.01	0.86	0.56	0.60 − 0.90	− 0.302^**^	**0.75**								
3. Psychological rumination	3.28	0.95	0.86	0.57	0.75 − 0.90	0.284^**^	− 0.212^**^	**0.75**							
4. Creativity	2.39	0.92	0.88	0.65	0.74 − 0.91	− 0.255^**^	0.299^**^	− 0.265^**^	**0.81**						
5. Emotional intelligence	3.18	1.29	0.98	0.79	0.59 − 0.98	− 0.253^**^	0.317^**^	-0.07	0.07	**0.89**					
6. Education Level	2.75	0.68	-	-		0.229^**^	-0.07	-0.01	0.05	-0.01	1.00				
7. Usage Frequency	2.83	1.14	-	-		0.11	0.05	0.12	-0.10	-0.05	-0.01	1.00			
8. Usage experience	2.26	0.78	-	-		-0.07	0.02	-0.05	-0.01	0.06	0.03	-0.02	1.00		
9. Number of friends	2.86	1.02	-	-		-0.03	-0.02	-0.12	0.01	-0.02	0.169^**^	0.05	0.10	1.00	
10. Job type	2.73	1.11	-	-		-0.11	0.05	− 0.153^*^	0.11	-0.02	-0.04	0.02	-0.06	0.06	1.00

Note: Diagonal cells have square roots of AVEs; *p = 0.05, **p = 0.01; ***p = 0.001

We further conducted additional tests followed by previous studies [40, 76] to address the potential effect of multicollinearity among all the constructs. In this test, we found the value of variance inflation factor (VIF) range between 1.10 and 1.21 is less than the threshold of 5 [91] there is no significant concern of multicollinearity in our dataset.

### Structural model

The tables illustrate the outcome of regression analysis, conducted to test our hypothesis. Our research suggests that SMO, psychological rumination, psychological safety, and emotional intelligence have a significant impact on employees’ creativity.

To analyze hypothesis relationships, we employed the AMOS version 24. The findings presented in Table 3 reveal the results of the hypothesis testing. Both Hypothesis 1 and Hypothesis 2 posit that SMO has a favorable impact on psychological rumination and negativity about the psychological safety experienced by users of social media. The outcomes displayed in Table 3 confirm these hypotheses, as they demonstrate a positive correlation between SMO and psychological rumination among social media users (β = 0.19, *P* < 0.001). Furthermore, our Hypothesis 2 is also sustained, indicating a negative association between SMO and the psychological safety of social media users (β = -0.21, *P* < 0.001). Consequently, both Hypothesis 1 and Hypothesis 2 are validated. In addition, the results also uphold Hypothesis 3 and Hypothesis 4, showing a negative relationship between psychological rumination and creativity (β = -0.18, *P* < 0.001)., as well as a positive connection between psychological safety and creativity among social media users (β = 0.24, *P* < 0.001).

**Table 3:** Results of a hypothesis test

	Β	S.E.	C.*R*.	*P*		β	S.E.	C.*R*.	*P*
Usage frequency to Employee creativity	-0.08	0.05	-1.57	0.12		-0.08	0.05	-1.57	0.12
Education to Employee creativity	0.10	0.08	1.29	0.20		0.10	0.08	1.29	0.20
Job type to Employee creativity	0.06	0.05	1.19	0.23		0.06	0.05	1.19	0.23
Usage experience to Employee creativity	-0.03	0.07	-0.46	0.65		-0.01	0.05	-0.27	0.79
Number of friends to Employee creativity	-0.01	0.05	-0.27	0.79		-0.03	0.07	-0.46	0.65
Social media ostracism to Rumination	0.19	0.04	4.62	***		0.32	0.06	5.07	***
Social media ostracism to Psychology safety	-0.21	0.04	-4.95	***		-0.28	0.06	-4.45	***
Psychology safety to Employee creativity	0.24	0.05	4.48	***		0.24	0.05	4.47	***
Rumination to Employee creativity	-0.18	0.06	-3.20	***		-0.18	0.06	-3.19	***
Emotional intelligence to Rumination	-	-	-	-		0.01	0.06	0.11	0.91
Emotional intelligence to Psychology safety	-	-	-	-		0.25	0.06	4.13	***
Interaction to Rumination	-	-	-	-		-0.12	0.05	-2.59	**
Interaction to Psychology safety	-	-	-	-		0.11	0.05	2.38	*

Table 4 shows, that Hypothesis 5 suggests that the link between SMO and employee creativity is mediated by psychological rumination and psychological safety. According to Baron and Kenny [92] following conditions must be fulfilled for mediation. First, the Mediator must be significantly affected by the independent variable. Second, the significant effect of the independent variable must be visible on the dependent variable. Third, the influence of the mediator on dependent variables must be significant. When all three conditions are fulfilled, the independent variable has less effect on the dependent variable in the presence of a mediator. The findings demonstrate significant relation between SMO and psychological rumination and psychological safety respectively (β = 0.19, *P* < 0.001, β = -0.21, *P* < 0.001). therefore, the first condition of mediation is fulfilled. SMO has a significant relation with employee creativity which fulfills the second condition (β = -0.16, *P* < 0.001). Psychological rumination and psychological safety significantly affect employee creativity (β = -0.20, *P* < 0.001, β = 0.22, *P* < 0.001), thereby supporting the third condition. The results indicate that psychological rumination and psychological safety partially mediate the link between SMO and employee creativity. Consequently, Hypothesis 5 is confirmed.

**Table 4:** Mediation effects

IV	M	DV	IV→DV	IV→M	IV + M→DV IV→DV	M→DV	
Social media ostracism	Rumination	Employee creativity	-0.16***	0.19***	-0.13***	-0.20***	Partial mediation
Social media ostracism	Psychology safety	Employee creativity	-0.16***	-0.21***	-0.12**	0.22***	Partial mediation

Note: Three step medthod is used to test mediation effect. Step 1: IV→M is significant. Step 2: IV→DV is significant. Step 3: IV + M→DV. (1) M has full mediation effect if IV is non-significance and M is significance. (2) If both IV and M are significance, then M has partial mediation effect

According to Hypothesis 6 & 7, the presence of emotional intelligence influences the connection between SMO and psychological rumination, as well as psychological safety. Specifically, with a high level of emotional intelligence, this relationship is weakened, while at a low level of emotional intelligence, it becomes stronger. This hypothesis is supported by the significant interaction term between SMO and emotional intelligence (β = -0.12, *P* < 0.01 & β = 0.11, *P* < 0.05) respectively.

**Fig. 2:**
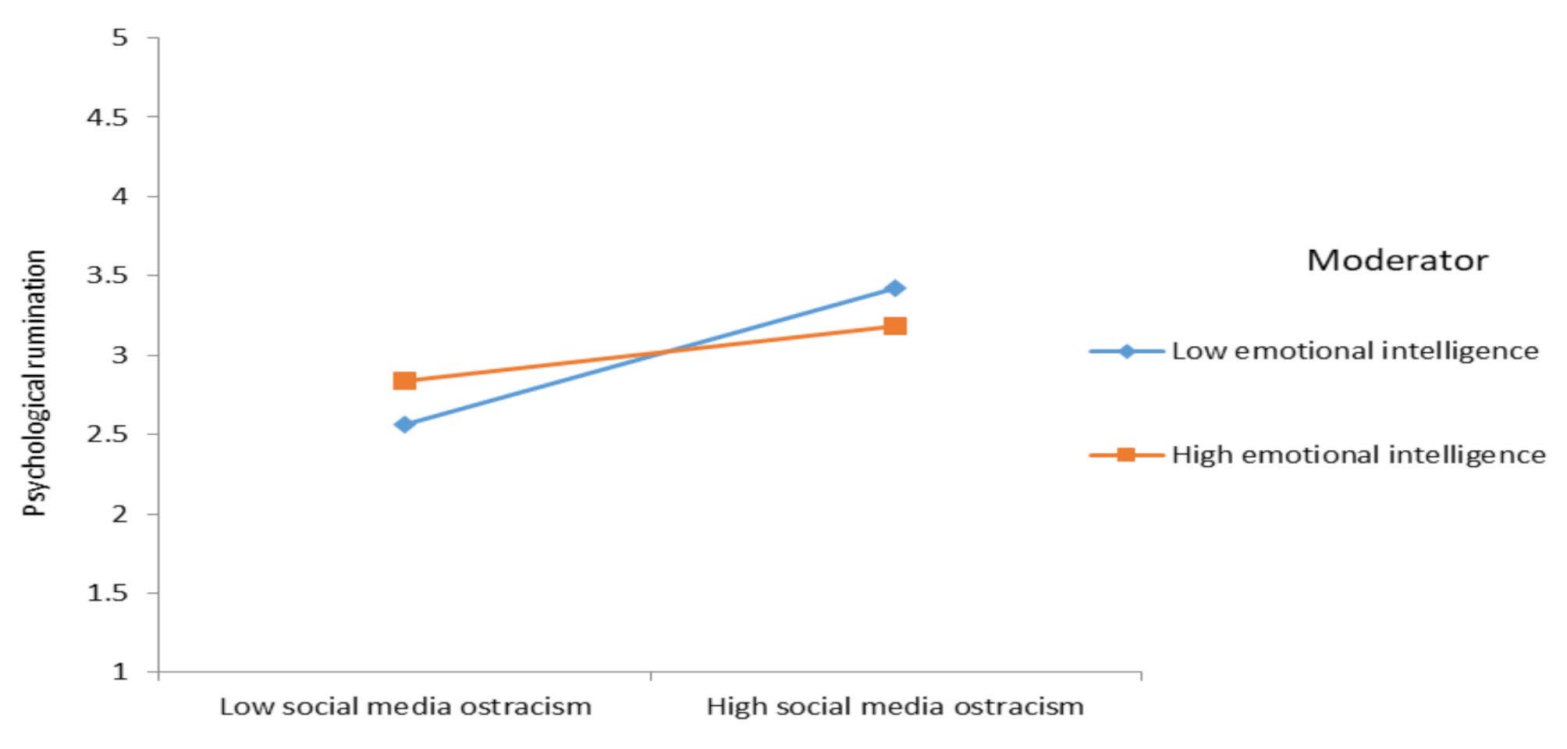
Interactive effect of SMO and emotional intelligence on psychological rumination

**Fig. 3:**
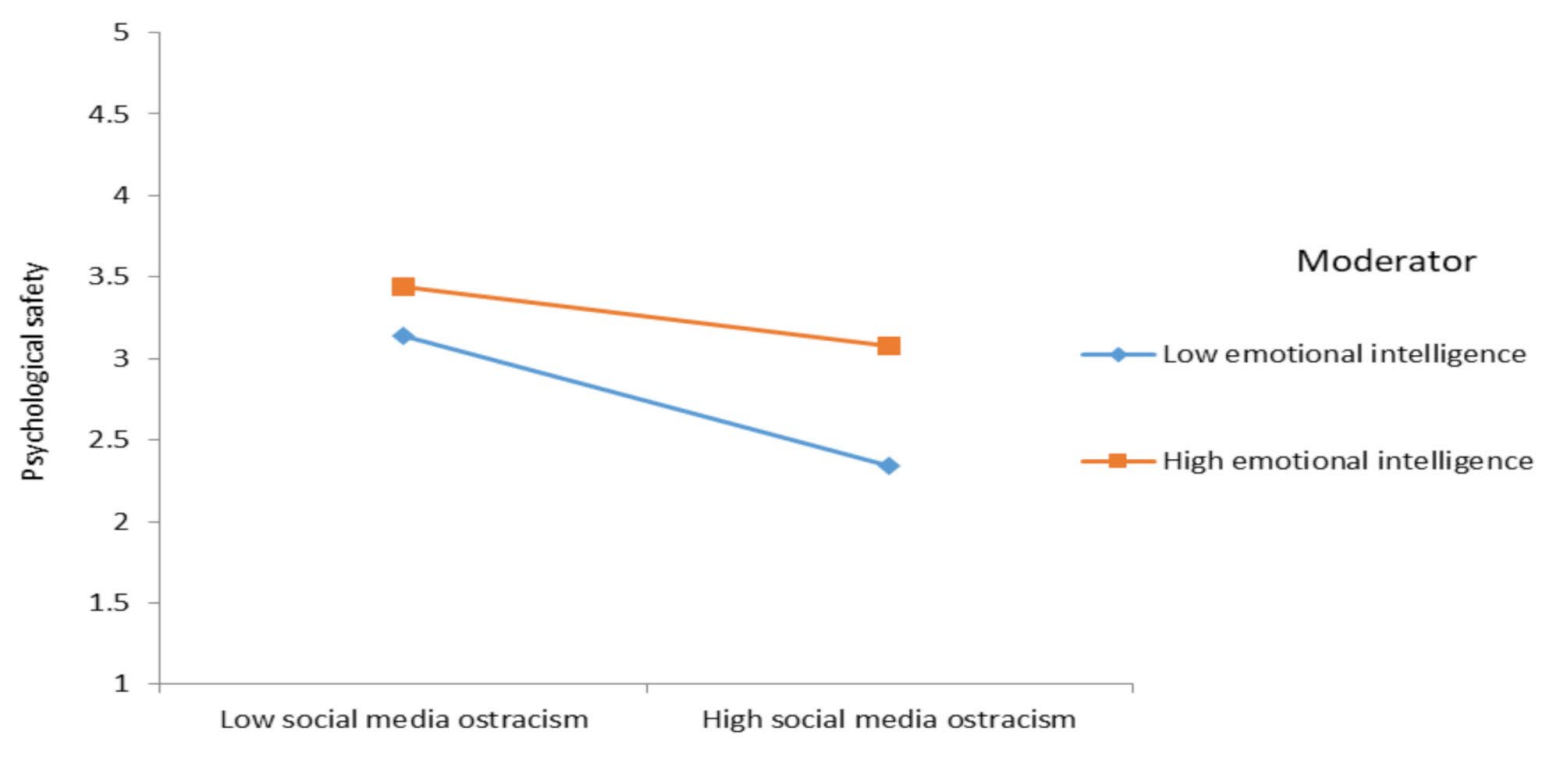
Interactive effect of SMO and emotional intelligence on psychological safety

**Fig. 4:**
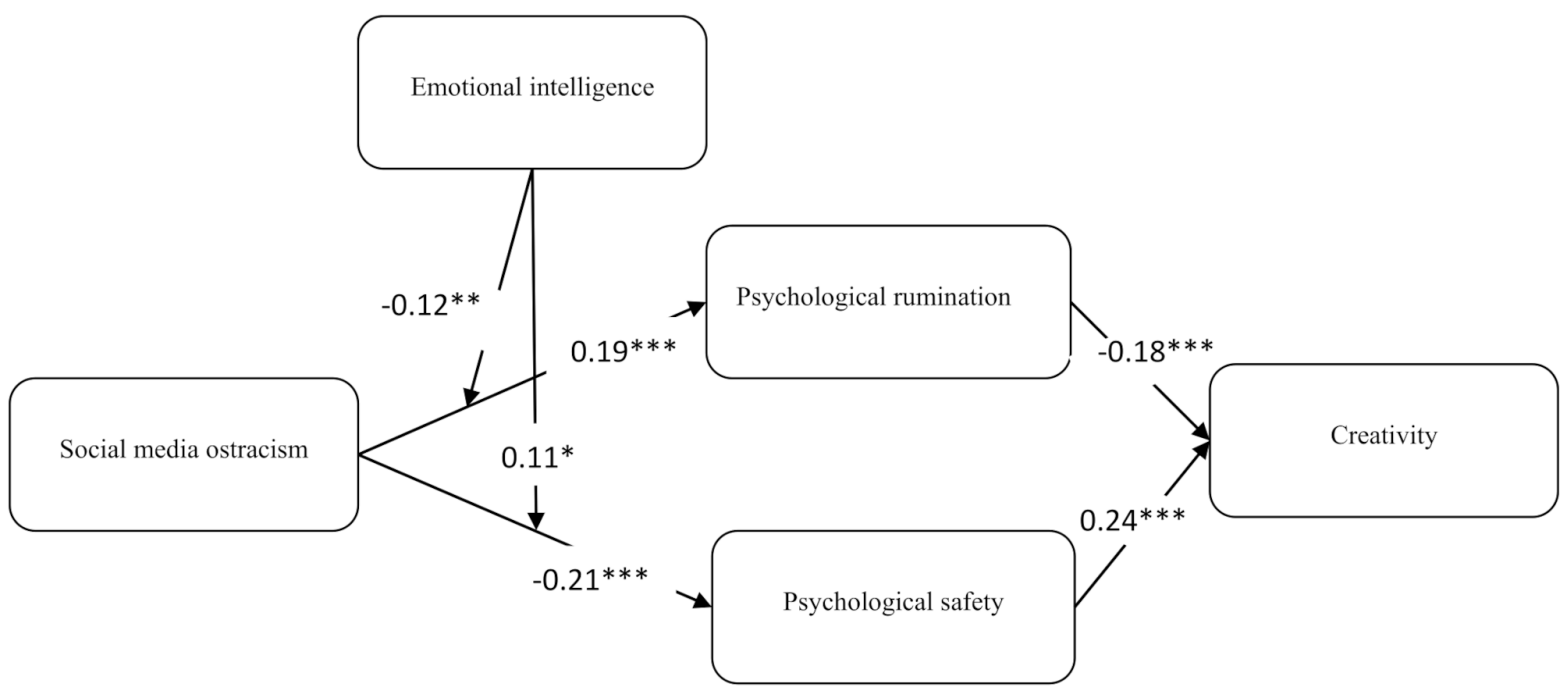
Results of AMOS analysis

To provide additional support for the moderation of hypotheses 6 and 7, the outcome of the post-hoc analysis following the approach outlined by [93] exposes the following: employees’ emotional intelligence, dampen the positive relationship between SMO psychological rumination of social media users (effect = 0.43, SE = 0.08, [0.26, 0.60], effect = 0.17, SE = 0.06, [0.03, 0.30]). Conversely, when social media users possess a high level of emotional intelligence, the negative relationship between SMO and psychological safety is relatively weak then those having low emotional intelligence level (effect = -0.39, SE = 0.09, [-0.57, -0.21], effect = -0.17, SE = 0.07, [-0.31, -0.03]). These findings are visually depicted in Fig. 2 and Fig. 3, reinforcing the post-hoc analysis’s contribution to providing auxiliary support for Hypotheses 6 and 7. Fig. 4 also shows hypothesis results.

## Discussion, implications, and limitations

### Discussion

The study attempts to find the effect of social media ostracism on employees’ creative performance, considering the mediating role of psychological rumination and psychological safety, and the moderating role of emotional intelligence with the support of ego depletion theory. We aspire to identify the narrative perspective of SMO and explore its effect on employees’ creativity. SMO decreases employees’ interaction with other employees and exerts psychological pressure and insecurity. Based on ego depletion theory, our study presents psychological rumination and psychological safety as mediators between SMO and creativity. Findings indicate that SMO depletes self-regulatory resources, leading to psychological rumination and psychological safety which impairs individual ability to perform creatively. It significantly affects organizations and social media users’ psychological condition. The findings of this study indicate that such critical feelings of ostracism enhance employees’ psychological rumination and decrease their psychological safety, thereby impacting their creativity. The novel aspect of this study lies in its timely approach to examining the complex relationship between SMO, employee psychology, and their creative performance. Furthermore, the ongoing research proposes that emotional intelligence in this relationship is particularly insightful. Previous studies have primarily investigated the effect of general social ostracism on individual psychology and performance [94, 95]. This study contributes to covering the existing gaps by groping the specific consequences of SMO on creativity, providing appreciated knowledge for organizations in managing the challenges associated with online interactions.

### Theoretical implication

Numerous theoretical support for social media literature is a part of this study. This research significantly contributes to the existing literature on social media by addressing a research gap related to the understanding of stressors in the context of social media. While previous studies have predominantly concentrated on exploring the various stressors in social media and their effects on workers and students [96, 97], the important stressor of ostracism has often been overlooked, with only a few studies considering its influence. However, it has been argued that exclusion is a prevalent phenomenon in various social settings, together with the realm of social media. Therefore, our research provides a platform for understanding social media stressors by specifically examining the impact of SMO on organizational creativity.

Our study builds upon earlier research that has explored the adverse effects of social media stressors by specifically investigating the impact of SMO on employee creativity. While existing literature has identified various stressors in the realm of social media [98, 99], it has often overlooked the crucial stressor of ostracism and its potential implications for employee attitudes and cognitive behavior. Particularly within the organizational context, the implications of SMO have been disregarded. By addressing these gaps, our research contributes to the expanding body of study on social media stressors and broadens the understanding of its consequences on employee creativity. By examining the experiences of employees who feel excluded, ignored, or rejected by their coworkers on ESM platforms, we uncover valuable empirical insights. These findings reveal that SMO prompts employees to adopt passive behaviors, leading them to refrain from sharing information, share ideas, contribute to team projects contributing content, and finally, they are less likely to actively participate in discussions. This lack of collaboration can impede the flow of information, limit knowledge sharing, and hinder problem-solving efforts.

This study builds on the framework of ego depletion theory [33] and identifies and confirms a significant pathway through which SMO influences cognitive outcomes, specifically concerning employees’ creativity. The research findings highlight two key factors, namely psychological rumination and psychological safety, as mediating variables in this process. The study reveals that SMO impacts employees’ creativity by depleting their self-control and well-being resources, as suggested by ego-depletion theory. This depletion of resources subsequently leads to increased psychological rumination and a decrease in psychological safety. Consequently, employees are less willing to engage in cognitive activities such as sharing ideas and actively participating in discussions. The outcomes of our study provide valuable empirical evidence that supports the mediation of psychological rumination and psychological safety in linking SMO to employees’ creativity. By establishing this link, our research contributes a fresh perspective on understanding the influence of SMO on employees’ creativity.

In our study, we not only identify a critical moderator that alleviates the adverse effects of SMO but also provide empirical evidence to support our findings. Furthermore, we found that individuals with high emotional intelligence are less likely to perceive SMO and experience fewer resource losses. This reduced impact on resource loss, in turn, diminishes the direct influence of SMO on psychological rumination and psychological safety, ultimately leading to a lesser impact on employees’ creativity. By investigating the moderating role of emotional intelligence, our study contributes to the existing literature by expanding our understanding of the moderating conditions that mitigate the effects of SMO on social media users. These findings shed light on the complex dynamics and provide deeper insights into the influence of SMO on individuals’ psychological and cognitive behavior.

Our study makes a valuable contribution to the existing framework of ego depletion theory. This theory has been widely employed in organizational behavior research to elucidate the impact of workplace-related factors on individual resources, and their subsequent influence on employees’ behavior and work performance [55, 69, 100–102]. However, we extended prior research on ego depletion theory by demonstrating that resource depletion is a crucial factor not only in traditional workplace settings but also among information system users [101]. Our findings indicate that self-regulatory resource depletion plays a significant role in the relationship between SMO and employees’ creativity. In other words, when resources are depleted, SMO negatively affects employees’ creative output.

With the implementation of a moderated mediation model, our study provides empirical evidence that aligns with the core principles of ego depletion theory. These findings strengthen the inclusivity of this theory and highlight its relevance in comprehending the dynamics of SMO and its impact on employees’ creativity.

### Managerial implication

This section of our study provides several practical implications for practitioners. The findings of our study expose that SMO is a key factor for employees to enthusiastically intermingle, make conversation, and share information using social media. Unluckily, this part has been neglected in the prior literature, in that way, managers are not much more aware of this situation that this social media stressor can diminish the value of social media acceptance in organizations. Particularly organizations approve social media (ESM) to communicate, interact, and share knowledge with internal and external members [5, 23, 41], our results drew attention toward the importance of SMO to enlighten its impact on employees’ psychological well-being and creativity. Hence, we advocate that organizations should flourish strategies to evade SMO by workers. A prior study recommended a series of trainings for social media users [103] and escalating workers’ face-to-face interaction to enlighten their understanding of social media usage [104, 105]. This practice is expected to hold back workers from SMO, thus normally mounting the efficacy of ESM, and ultimately increasing their creativity. Based on our findings workers who experience SMO are likely to encounter psychological pressure, consequentially declining their cognitive capability.

Results of our research also point out that employees who perceive SMO are likely to encounter a shrinking of their self-control and willpower resources, resulting in psychological rumination and decreasing their psychological safety, consequently affecting their creativity. Given our result of the mediating effect of psychological rumination and psychological safety linking SMO with employees’ creativity, we advocate that organizations reduce psychological rumination and enhance psychological safety. One possible suggestion for organizations is to create a general atmosphere, offer supplementary social resources, and provide opportunities to individuals with high emotional intelligence to help workers achieve the required job resources.

In addition, our results disclose that workers with high-level emotional intelligence are less susceptible to SMO. Our results recommend that the HR department consider emotional intelligence in hiring procedures to facilitate employees handling stressors. Therefore, we auxiliary recommend that organizations consider interference during employment and work (e.g. enrollment tests, training programs, and social support).

### Limitations

It is important to acknowledge the limitations of this study, as they provide opportunities for future research. Firstly, our data collection was conducted exclusively in China. While China serves as a representative example of an emerging economy, it possesses distinct cultural, behavioral, and value-related characteristics that may introduce biases to our findings. For instance, Chinese organizations often rely heavily on personal relationships for task completion, which may differ from Western contexts in terms of instrumental value. Consequently, the impact of ostracism may be more obvious in the Chinese context. Therefore, it is recommended that future studies apply the conceptual framework utilized in this study to other countries with diverse economic, political, and cultural environments to examine the generalizability of our findings.

Second, one important limitation of our study is the potential for common method bias due to the data collection method. We acknowledge that collecting data from the same source for all variables can introduce the possibility of common method bias [106]. Addressing this concern, we employed multiple methods and conducted rigorous tests to comprehensively evaluate the effect of common method bias in our study. We aimed to mitigate the impact of common method bias on our results. In summary, while we made efforts to address common method bias, the data collection method used in this study has limitations. Future research should employ alternative data collection methods to further mitigate the potential influence of common method bias and enhance the generalizability of the findings. For instance, gathering data from colleagues, subordinates, and co-workers in addition to the original data source would be advantageous when examining creativity.

Third, future research can also investigate the role of individual-level variables, such as personality traits or cultural orientations, in moderating the effects of SMO. Understanding how individual differences interact with contextual factors can enhance our understanding of the complex dynamics involved in SMO.

Lastly, another important limitation to consider is that while our theoretical model suggests that SMO is associated with creative performance through psychological rumination and psychological safety, there may be alternative theoretical mechanisms that explain this relationship. For instance, previous studies have highlighted the concept of social media stressors leading to social media fatigue and anxiety [11, 107]. Therefore, it is recommended that future studies explore and examine our theoretical model using alternative variables and mechanisms.

## Conclusion

This study investigates how SMO influences employees’ creativity. The mediating role of psychological rumination and psychological safety were investigated based on ego depletion theory. All hypotheses were supported, showing that the psychological condition of employees is very important in transmitting the consequence of SMO on employees’ creativity. Furthermore, the moderation effect of emotional intelligence plays a vital role in handling social media stressors and ultimately leads to employees’ creativity. This study adds value to SMO literature by integrating ego-depletion theory, using the mediation moderation model to discuss the outcome of SMO on employees’ creativity. Additionally, managers are advised to check employees’ psychological conditions and train them to handle stressors that would not affect on their creativity.

## Electronic supplementary material

Below is the link to the electronic supplementary material.

Supplementary Material 1

Supplementary Material 2

Supplementary Material 3

Supplementary Material 4

Supplementary Material 5

Supplementary Material 6

## Data Availability

The data that support the findings of this study are available upon reasonable request from the corresponding author Muhammad Waqas Amin. The data are not publicly available because it contains information that could compromise the privacy of the research participants.
