# Supplementary material for: Social media ostracism and creativity: moderating role of emotional intelligence
Source: BMC Psychol. 2024 Sep 13;12:484. doi: 10.1186/s40359-024-01985-2 (PMC11401364; doi:10.1186/s40359-024-01985-2)
Supplement: Supplementary file 2 — Supplementary Material 2 [file 40359_2024_1985_MOESM2_ESM.pdf]

Model Fit Summary

CMIN

Model	NPAR	CMIN	DF	P	CMIN/DF
Default model	146	1552.921	714	.000	2.175
Saturated model	860	.000	0		
Independence model	80	15225.448	780	.000	19.520

Baseline Comparisons

Model	NFI Delta1	RFI rho1	IFI Delta2	TLI rho2	CFI
Default model	.898	.889	.942	.937	.942
Saturated model	1.000		1.000		1.000
Independence model	.000	.000	.000	.000	.000

Parsimony-Adjusted Measures

Model	PRATIO	PNFI	PCFI
Default model	.915	.822	.862
Saturated model	.000	.000	.000
Independence model	1.000	.000	.000

NCP

Model	NCP	LO 90	HI 90
Default model	838.921	728.980	956.579
Saturated model	.000	.000	.000
Independence model	14445.448	14047.961	14849.325

FMIN

Model	FMIN	F0	LO 90	HI 90

Default model	1844.921	1904.189
Saturated model	1720.000	2069.109
Independence model	15385.448	15417.923

ECVI

Model	ECVI	LO 90	HI 90	MECVI
Default model	7.592	7.140	8.076	7.836
Saturated model	7.078	7.078	7.078	8.515
Independence model	63.315	61.679	64.977	63.448

HOELTER

Model	HOELTER .05	HOELTER .01
Default model	122	126
Independence model	14	14
