# Supplementary material for: Social media ostracism and creativity: moderating role of emotional intelligence
Source: BMC Psychol. 2024 Sep 13;12:484. doi: 10.1186/s40359-024-01985-2 (PMC11401364; doi:10.1186/s40359-024-01985-2)
Supplement: Supplementary file 3 — Supplementary Material 3 [file 40359_2024_1985_MOESM3_ESM.pdf]

Estimates (Group number 1 - Default model)

Scalar Estimates (Group number 1 - Default model)

Maximum Likelihood Estimates

Regression Weights: (Group number 1 - Default model)

			Estimate	S.E.	C.R.	P	La
RUMI	<---	SMO	.188	.041	4.615	***	
Psy	<---	SMO	-.212	.043	-4.945	***	
Crea	<---	Psy	.242	.054	4.481	***	
Crea	<---	RUMI	-.183	.057	-3.195	.001	
Crea	<---	Usage_Frequency	-.075	.048	-1.567	.117	
Crea	<---	EducationLevel	.103	.080	1.288	.198	
Crea	<---	Job_type	.058	.049	1.192	.233	
Crea	<---	Usage_experience	-.032	.070	-.457	.648	
Crea	<---	Number_of_Friends	-.014	.053	-.270	.787	

Variances: (Group number 1 - Default model)

	Estimate	S.E.	C.R.	P	Label
SMO	2.058	.187	11.023	***	
Usage_Frequency	1.287	.117	11.023	***	
EducationLevel	.460	.042	11.023	***	
Job_type	1.220	.111	11.023	***	
Usage_experience	.601	.055	11.023	***	
Number_of_Friends	1.030	.093	11.023	***	
e3	.827	.075	11.023	***	
e4	.918	.083	11.023	***	
e5	.713	.065	11.023	***	
