# Supplementary material for: Social media ostracism and creativity: moderating role of emotional intelligence
Source: BMC Psychol. 2024 Sep 13;12:484. doi: 10.1186/s40359-024-01985-2 (PMC11401364; doi:10.1186/s40359-024-01985-2)
Supplement: Supplementary file 4 — Supplementary Material 4 [file 40359_2024_1985_MOESM4_ESM.pdf]

Estimates (Group number 1 - Default model)

Scalar Estimates (Group number 1 - Default model)

Maximum Likelihood Estimates

Regression Weights: (Group number 1 - Default model)

			Estimate	S.E.	C.R.	P	La
Psy	<---	ZEmInt	.251	.061	4.125	***	
Psy	<---	ZSMO	-.282	.063	-4.448	***	
RUMI	<---	ZEmInt	.007	.060	.112	.911	
RUMI	<---	ZSMO	.315	.062	5.068	***	
Psy	<---	iNT	.110	.046	2.382	.017	
RUMI	<---	iNT	-.118	.045	-2.592	.010	
Crea	<---	RUMI	-.183	.057	-3.188	.001	
Crea	<---	Psy	.242	.054	4.471	***	
Crea	<---	Usage_Frequency	-.075	.048	-1.567	.117	
Crea	<---	EducationLevel	.103	.080	1.288	.198	
Crea	<---	Job_type	.058	.049	1.192	.233	
Crea	<---	Number_of_Friends	-.014	.053	-.270	.787	
Crea	<---	Usage_experience	-.032	.070	-.457	.648	

Covariances: (Group number 1 - Default model)

			Estimate	S.E.	C.R.	P	Label
ZEmInt	<-->	ZSMO	-.252	.066	-3.824	***	
ZEmInt	<-->	iNT	-.031	.085	-.366	.715	
ZSMO	<-->	iNT	.367	.088	4.180	***	

Variances: (Group number 1 - Default model)

	Estimate	S.E.	C.R.	P	Label
ZEmInt	.996	.090	11.023	***	
