# Supplementary material for: Social media ostracism and creativity: moderating role of emotional intelligence
Source: BMC Psychol. 2024 Sep 13;12:484. doi: 10.1186/s40359-024-01985-2 (PMC11401364; doi:10.1186/s40359-024-01985-2)
Supplement: Supplementary file 5 — Supplementary Material 5 [file 40359_2024_1985_MOESM5_ESM.pdf]

Model Validity Measures

Validity Analysis

	CR	AVE	MSV	MaxR(H)	EIntell	SMOST	PsySaf	Rumm	Creat
**EIntell**	0.984	0.791	0.635	0.985	**0.889**				
**SMOST**	0.974	0.792	0.635	0.978	-0.797***	**0.890**			
**PsySaf**	0.866	0.566	0.352	0.881	0.593***	-0.474***	**0.752**		
**Rumm**	0.869	0.573	0.245	0.883	-0.495***	0.380***	-0.240**	**0.757**	
**Creat**	0.883	0.658	0.099	0.910	0.283***	-0.315***	0.183*	-0.229**	**0.811**

Validity Concerns

No validity concerns here.

HTMT Analysis

	EIntell	SMOST	PsySaf	Rumm	Creat
**EIntell**					
**SMOST**	0.780				
**PsySaf**	0.609	0.471			
**Rumm**	0.502	0.374	0.251		
**Creat**	0.286	0.340	0.205	0.256	

HTMT Warnings

There are no warnings for this HTMT analysis.

References

Significance of Correlations:
† p < 0.100
* p < 0.050
** p < 0.010
*** p < 0.001

Thresholds From:
Hu, L., Bentler, P.M. (1999), "Cutoff Criteria for Fit Indexes in Covariance Structure Analysis: Conventional Criteria Versus New Alternatives" SEM vol. 6(1), pp. 1-55.
Henseler, J., C. M. Ringle, and M. Sarstedt (2015). A New Criterion for Assessing Discriminant Validity in Variance-based Structural Equation Modeling, Journal of the Academy of Marketing Science, 43 (1), 115-135.
Thresholds are 0.850 for strict and 0.900 for liberal discriminant validity.

--If you would like to cite this tool directly, please use the following: Gaskin, J., James, M., and Lim, J. (2019), "Master Validity Tool", AMOS Plugin. Gaskination's StatWiki.
