# Supplementary material for: Social media ostracism and creativity: moderating role of emotional intelligence
Source: BMC Psychol. 2024 Sep 13;12:484. doi: 10.1186/s40359-024-01985-2 (PMC11401364; doi:10.1186/s40359-024-01985-2)
Supplement: Supplementary file 6 — Supplementary Material 6 [file 40359_2024_1985_MOESM6_ESM.pdf]

Appendix

Variable	ID	Measurement	Source
**Social Media Ostracism**	SMO1	My friends ignore me on enterprise social media	Ali A, Wang H, Gong M, Mehmood K. Conservation of resources theory perspective of social media ostracism influence on lurking intentions. Behaviour & Information Technology. 2024;43(1):212-29.
	SMO2	My connection goes offline when I use enterprise social media	
	SMO3	My greeting goes unanswered on enterprise social media	
	SMO4	I involuntarily feel alone on enterprise social media	
	SMO5	My social media connection avoid me on enterprise social media	
	SMO6	I noticed my friends would not look at my posts on enterprise social media	
	SMO7	My friends on enterprise social media shut me out of the conversation	
	SMO8	My enterprise social media friends refused to talk with me	
	SMO9	My friends on enterprise social media treat me as if I am not online	
	SMO10	My enterprise social media friends don't invite me when the play online games	

**Psychological rumination**	Psy R1	I had trouble doing other things because different thoughts are coming into my mind	McCullough ME, Bono G, Root LM. Rumination, emotion, and forgiveness: three longitudinal studies. Journal of personality and social psychology. 2007;92(3):490.
	Psy R2	I wonder why I have these problems and others don't	
	Psy R3	I found myself playing with thoughts over and over in my mind	
	Psy R4	I think about my failures	
	Psy R5	My mental energy is running low	
**Psychological safety**	Psy S1	Members of this organization are able to bring up problems and tough issues	Edmondson A. Psychological safety and learning behavior in work teams. Administrative science quarterly. 1999;44(2):350-83.
	Psy S2	People in this organization sometimes reject others for being different	
	Psy S3	It is difficult to ask other members of this organization for help	
	Psy S4	No one in this organization would deliberately act in a way that undermines my efforts	
	Psy S5	Working with members of this organization, my unique skills and talents are valued and utilized	

**Emotional Intelligence**	EI1	I have a good sense of why I have certain feelings most of the time.	Wong C-S, Law KS. The effects of leader and follower emotional intelligence on performance and attitude: An exploratory study. Leadership Perspectives: Routledge; 2017. p. 97-128.
	EI2	I have good understanding of my own emotions.	
	EI3	I really understand what I feel.	
	EI4	I always know whether or not I am happy	
	EI5	I always know my friends' emotions from their behavior.	
	EI6	I am a good observer of others' emotions.	
	EI7	I am sensitive to the feelings and emotions of others.	
	EI8	I have good understanding of the emotions of people around me	
	EI9	I always set goals for myself and then try my best to achieve them.	
	EI10	I always tell myself I am a competent person	
	EI11	I am a self-motivated person.	
	EI12	I would always encourage myself to try my best.	

	EI13	I am able to control my temper and handle difficulties rationally.	
	EI14	I am quite capable of controlling my own emotions.	
	EI15	I can always calm down quickly when I am very angry.	
	EI16	I have good control of my own emotions.	
**Creativity**	Cr1	This employee identifies opportunities for new ways of dealing with work	Jaiswal NK, Dhar RL. Transformational leadership, innovation climate, creative self-efficacy and employee creativity: A multilevel study. International journal of hospitality management. 2015;51:30-41.
	Cr2	This employee seeks new ideas and ways to solve problems	
	Cr3	This employee generates novel but operable work-related ideas	
	Cr4	This employee demonstrates originality in his/her work	
